# Good and Bad News about Ebola

**DOI:** 10.1371/journal.pntd.0003509

**Published:** 2015-03-12

**Authors:** A. Townsend Peterson

**Affiliations:** Biodiversity Institute, University of Kansas, Lawrence, Kansas, United States of America

Filoviruses have been known to science and medicine since 1967 (Marburg virus) and 1976 (Ebola virus). Since then, more than 30 independent transmission events have been documented between the zoonotic world and the human world, and more than 2,300 human cases have been recorded [1]. The outbreak that began in West Africa in 2014, however, has changed the equation considerably; although it began in a remote district of Guinea [2], it has magnified massively, caused large numbers of cases and deaths in three West African countries, dispersed into three other West African countries, and “jumped” now several times out of Africa to North America and Europe. That is the bad news, which is measured in human mortality and suffering.

So what good news could come out of this massive outbreak? Well, finally, Ebola is seeing intense attention in terms of research and funding. Already, several vaccines and treatments have been fast-tracked for testing and distribution and are producing promising results with the relatively few patients who have been able to access them. Surely this research has not been generated just in the past few months; rather, these steps forward derive from years of “low priority,” minimally funded research that had made significant progress [3], laying a foundation for rapid advances [4]. Clearly, the reason why no Ebola vaccine was already in hand was that research funders and pharmaceutical industry investors had not made it a priority, likely because the disease was not perceived as a threat, or as a good investment.

Take into consideration the mission statements of the two United States government entities that are most relevant to this commentary. The mission of the Centers for Disease Control and Prevention (CDC) is “… to protect America from health, safety and security threats, both foreign and in the U.S. Whether diseases start at home or abroad, are chronic or acute, curable or preventable, human error or deliberate attack, CDC fights disease and supports communities and citizens to do the same…” That of the National Institutes of Health (NIH) is “… to develop, maintain, and renew scientific human and physical resources that will ensure the Nation's capability to prevent disease; to expand the knowledge base in medical and associated sciences in order to enhance the Nation's economic well-being and ensure a continued high return on the public investment in research…” Note, in both cases, the focus is on health and disease prevention for Americans.

Now, however, a very different dynamic is developing. In a recent hearing of the Oversight and Government Reform Committee of the US House of Representatives, Representative Jim Jordan (Republican, Ohio) confronted Dr. Nicole Lurie, Assistant Secretary of Health and Human Services for Emergency Preparedness and Response, with a $38 million list of NIH expenditures that he considered unnecessary (e.g., fruit and vegetable puppet shows stressing healthy menus for children and studies encouraging senior citizens to join choirs). He asked, “wouldn’t we now have two Ebola vaccines if we hadn’t spent money on those?” [5]. The usual rhetoric about unnecessary grant spending and the lack of understanding of the importance of diet and activity in maintaining health both aside, Rep. Jordan missed the fact that Ebola was very low on the list of priorities until it threatened to spread broadly to the US this year and never would have seen any benefit were those $38 million to have been freed up, or even if the NIH budget were to have increased substantively, as is badly needed.

The simple fact is that the world has become very small in recent decades. Connectivity via rapid travel on local, regional, and global scales makes epidemic spread massively efficient [6]. This global linkage means that a person almost anywhere in the world can be in the US or Europe within 48 hours, and thus that no disease outbreak anywhere can be considered as unlinked to the US or Europe, at least potentially [7]. The implications for disease control and prevention, and particularly for taking care of “American” interests in that regard, are immense—the number and variety of diseases that may come into play are considerable.

**Fig 1:**
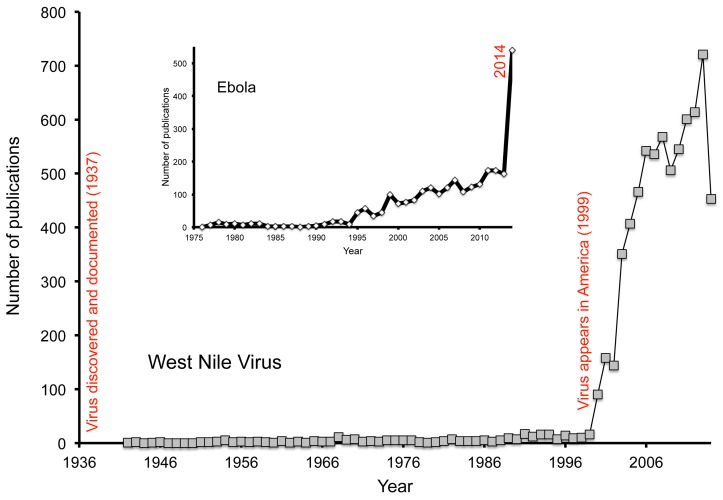
Summary of research attention to West Nile virus to present, in terms of number of publications with the term “West Nile virus” in Web of Science; inset shows parallel trends for Ebola research attention.

In this regard, the hemispheric or global spread of a series of diseases comes into a different light: Severe Acute Respiratory Syndrome (SARS), West Nile virus, and chikungunya all are examples [8]. Note that each of these diseases was unknown to or ignored entirely by the pharmaceutical industry, and slightly less completely by the research funding and research communities. Only when they spread into the US and European realm did these diseases see intense research attention—the arrival of West Nile virus in North America [9] is an excellent example: a total of 278 publications accumulated from 1942 through 1998, but yearly numbers of publications averaged 420 once the virus arrived (Fig. 1). Ebola is already following a similar trajectory after this (tragic) banner year of 2014, as can be appreciated from the inset in Fig. 1.

So what hope is there for the numerous neglected diseases, particularly in the Tropics? Many of them never get identified and described by scientists (see, e.g., the case of Lujo virus [10]) because they are not spectacular in their effects or because they do not occur in areas with good medical diagnostic facilities; those that are known are neglected in the sense that no pharmaceutical company would invest in a cure, a vaccine, or even intense research on them. Such neglected diseases include Chagas disease, human African trypanosomiasis, the leishmaniases, echinococcosis, lymphatic filariasis, onchocerciasis, schistosomiasis, Buruli ulcer, and many others [11]. Although research on neglected tropical diseases is supported by several US government programs (e.g., President's Malaria Initiative, President's Emergency Plan for AIDS Relief, and USAID’s Neglected Tropical Disease Program), as well as by several international and nongovernmental efforts (e.g., Global Fund to Fight AIDS, Tuberculosis and Malaria; Bill & Melinda Gates Foundation; Children’s Investment Fund Foundation; World Bank Group), clearly, the challenge is costlier than present resources can manage [12].

If the present situation with Ebola is to offer any lessons, they are that the only hope for serious investment in reducing the incidence and impact of such diseases is via spread to developed countries. Once such spread occurs, research and pharmaceutical investment will most likely follow. Ebola is a positive example, and clearly Ebola research will enter a new phase of progress, innovation, funding, production of key pharmaceuticals, and improved care, hopefully for all who might be infected by this virus.

In effect, what Ebola did was to cross the line between being a “neglected tropical disease” and being an “emerging infection.” The former set of diseases collectively exert an enormous burden in the developing world that may be constant or episodic, but are rather ubiquitous in those regions, affecting the affluent only when they venture into those regions [13,14]. The latter, on the other hand, are much less predictable, but garner more immediate attention on the world scene, precisely because they may affect affluent countries. How many other neglected diseases must await this process of spread to affluent regions and infection of affluent people, making the transition from neglected tropical disease to emerging infection, before they also will see investment and innovation?
